# Description and classification of echolocation clicks of Indian Ocean humpback (*Sousa plumbea*) and Indo-Pacific bottlenose (*Tursiops aduncus*) dolphins from Menai Bay, Zanzibar, East Africa

**DOI:** 10.1371/journal.pone.0230319

**Published:** 2020-03-13

**Authors:** Liangliang Yang, Matt Sharpe, Andrew J. Temple, Narriman Jiddawi, Xiaomei Xu, Per Berggren

**Affiliations:** 1 School of Natural and Environmental Sciences, Newcastle University, Newcastle upon Tyne, England, United Kingdom; 2 Key Laboratory of Underwater Acoustic Communication and Marine Information Technology of the Ministry of Education, College of Ocean and Earth Sciences, Xiamen University, Xiamen, China; 3 Institute of Fisheries Research Zanzibar, Ministry of Agriculture, Natural Resources, Livestock and Fisheries, Zanzibar, Tanzania; Wildlife Conservation Society Canada, CANADA

## Abstract

Passive acoustic monitoring (PAM) is a powerful method to study the occurrence, movement and behavior of echolocating odontocetes (toothed whales) in the wild. However, in areas occupied by more than one species, echolocation clicks need to be classified into species. The present study investigated whether the echolocation clicks produced by small, at-risk, resident sympatric populations of Indian Ocean humpback dolphin (*Sousa plumbea*) and Indo-Pacific bottlenose dolphin (*Tursiops aduncus*) in Menai Bay, Zanzibar, East Africa, could be classified to allow species specific monitoring. Underwater sounds of *S*. *plumbea* and *T*. *aduncus* groups were recorded using a SoundTrap 202HF in January and June-August 2015. Eight acoustic parameters, i.e. -10 dB duration, peak, centroid, lower -3 and lower -10 dB frequencies, and -3 dB, -10 dB and root-mean-squared bandwidth, were used to describe and compare the two species’ echolocation clicks. Statistical analyses showed that *S*. *plumbea* clicks had significantly higher peak, centroid, lower -3 and lower -10 dB frequencies compared to *T*. *aduncus*, whereas duration and bandwidth parameters were similar for the two species. Random Forest (RF) classifiers were applied to determine parameters that could be used to classify the two species from echolocation clicks and achieved 28.6% and 90.2% correct species classification rates for *S*. *plumbea* and *T*. *aduncus*, respectively. Both species were classified at a higher rate than expected at random, however the identified classifiers would only be useful for *T*. *aduncus* monitoring. The frequency and bandwidth parameters provided most power for species classification. Further study is necessary to identify useful classifiers for *S*. *plumbea*. This study represents a first step in acoustic description and classification of *S*. *plumbea* and *T*. *aduncus* in the western Indian Ocean region, with potential application for future acoustic monitoring of species-specific temporal and spatial occurrence in these sympatric species.

## Introduction

Passive acoustic monitoring (PAM) is a powerful technique to study the occurrence, movement and behavior of odontocetes (toothed whales) in the wild [1–4]. Compared to traditional visual surveys for odontocetes, PAM detections are not affected by visibility, weather conditions, or human observer bias and may be used for long-term monitoring with minimal disturbance to the study animals [5]. Odontocetes predominantly use two types of sounds; tonal frequency-modulated sounds for communication, e.g. whistles, [6]; and high frequency pulsed clicks, e.g. echolocation clicks for navigation, orientation and prey detection [7], and burst pulses for communication [8]. Whistles are highly variable at an individual level [9] whereas echolocation clicks (here on referred to as “clicks”), are more consistent and can be used for species classification [10–13]. However, some sympatric species of odontocetes produce similar clicks which can limit the effectiveness of PAM for species-specific studies, as acoustic species classification can be challenging [14].

Acoustic parameters of odontocete clicks vary depending on their sound production morphology [14], with some species having the ability to optimize their clicks within the context of the specific habitat [15]. These parameters are further influenced by sound propagation [11], off-axis effects [16] and differences in recording systems [14]. Previous research has successfully classified clicks for some species groups, such as narrowband high frequency clicks of phocoenids [17] and non-whistling delphinids [10], and at species-specific level e.g. sperm whales (*Physeter microcephalus*) [18], pygmy sperm whales (*Kogia breviceps*) [19], Cuvier’s beaked whales (*Ziphius cavirostris*) [20], Blainville’s beaked whales (*Mesoplodon densirostris*) [21] and Franciscana river dolphins (*Pontoporia blainvillei*) [22]. However, classification of clicks for delphinids, especially sympatric species, has proved difficult due to overlaps in some acoustic parameters among species [14, 23, 24].

If the species identity is known when collecting acoustic data, then supervised classification techniques can be developed and employed to attempt species classification using only click data. Supervised machine learning techniques, such as logic-based techniques (e.g. decision tree and rule-based classifiers), perception-based techniques (e.g. neural networks) and statistical learning algorithms (e.g. Bayesian networks and instance-based learning), perform well in processing complex input tasks and may improve decision-making and prediction of unlabeled samples [25]. Considerable efforts have been devoted to analyzing the species-specific aspects of sympatric delphinid clicks using various supervised machine learning methods [26, 27]. For example, clicks of melon-headed whales (*Peponocephala electra*), common bottlenose (*Tursiops truncatus*) and Gray’s spinner (*Stenellla longirostris longirostris*) dolphins were separated using spectral parameters and discriminant function analysis providing 93%, 75% and 54% correct classification rates for the three delphinid species, respectively [14]. Furthermore, clicks of seven delphinid species, striped dolphin (*Stenella coeruleoalba*), long-beaked common dolphin (*Delphinus capensis*), short-beaked common dolphin (*Delphinus delphis*), Risso’s dolphin (*Grampus griseus*), Pacific white-sided dolphin (*Lagenorhynchus obliquidens*), pilot whale (*Globicephala macrorhynchus*) and killer whale (*Orcinus orca*), off the coasts of Washington, Oregon and California were classified using the Random Forest classification model with overall correct classification score of 49%, which was significantly greater than that expected by chance for the seven species (14%) [12].

**Fig 1:**
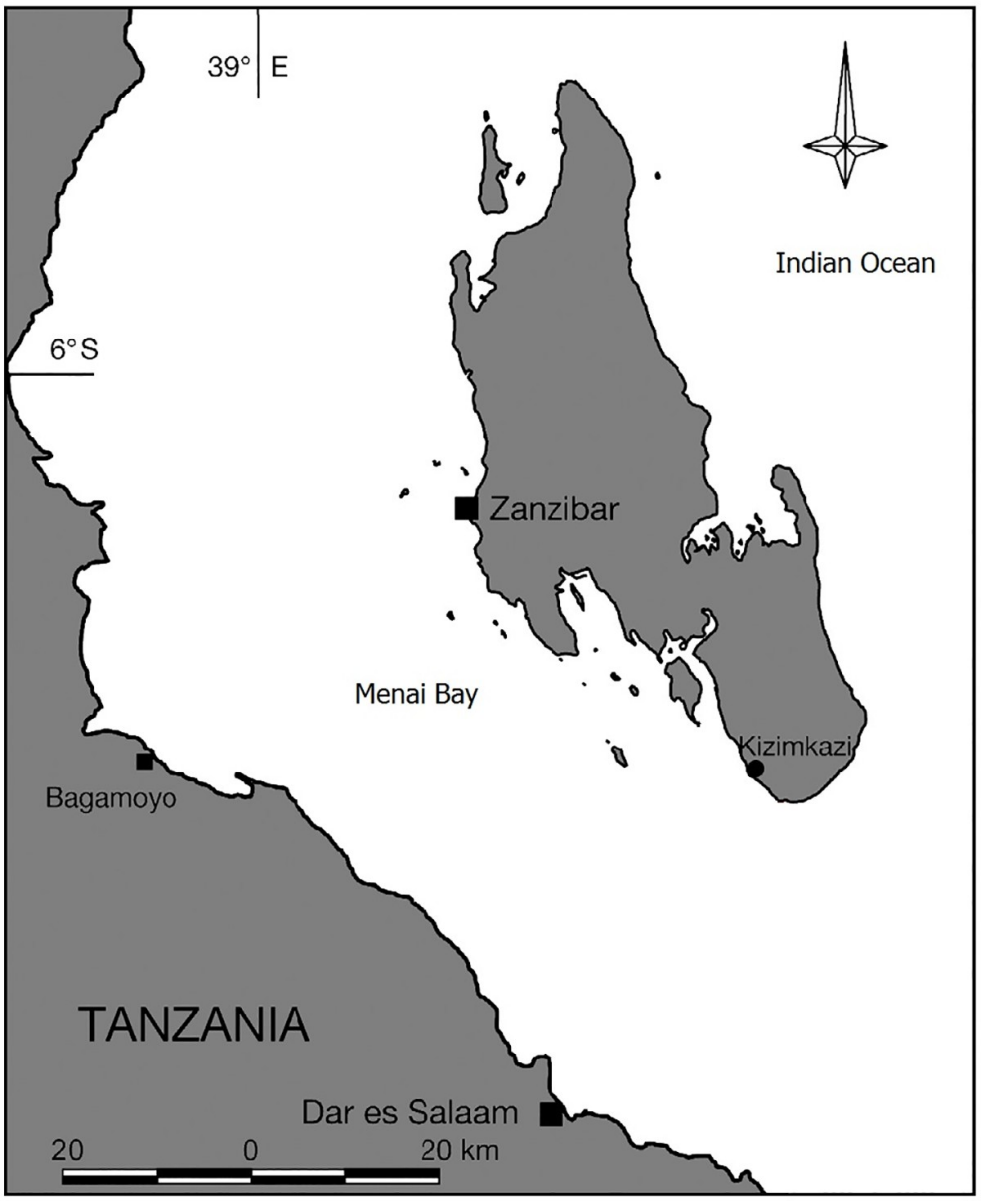
Map of study area. Menai Bay study area (6°31′S to 6°17′S, 39°11′E to 39°33′E) off the southwest coast of Zanzibar, East Africa, where recordings of *S*. *plumbea* and *T*. *aduncus* echolocation clicks were conducted in 2015.

Eight species of delphinids have been identified around Zanzibar, East Africa [28, 29], but only the Indian Ocean humpback (*Sousa plumbea*) and the Indo-Pacific bottlenose dolphin (*T*. *aduncus*) are resident in Menai Bay off the southwest coast (Fig 1). Boat surveys using photographic identification and capture-recapture analyses have estimated population sizes of 19 (95% CI 14–25) *S*. *plumbea* and 136 (95% CI 124–172) *T*. *aduncus* in the southern portion of the Menai Bay [30]. Both species are usually observed in small groups of 5–10 individuals, but social and foraging groups may be larger [30, 31]. *S*. *plumbea* is generally distributed closer to shore than *T*. *aduncus* in areas where the two species distributions overlap [32]. This is also true for Menai Bay, however the two species distributions overlap and are frequently encountered together in mixed-species groups [30]. Both species in Menai Bay are threatened by unsustainable fisheries bycatch [33, 34] and dolphin ecotourism [35–37].

The vocal repertoire of both *S*. *plumbea* and *T*. *aduncus* includes whistles and clicks, although to date only whistles of *T*. *aduncus* from Zanzibar have been described in detail [38]. A recent PAM study conducted in Menai Bay demonstrated broad scale spatio-temporal occurrence patterns for the delphinids [39]. However, that study was unable to acoustically classify the two species and therefore unable to assess species-specific spatio-temporal patterns, thus limiting the applications of the results. Yet, there is potential for species separation, with small but significant differences in some broadband click parameters demonstrated between *T*. *aduncus* and Australian humpback dolphins (*S*. *sahulensis*) [23], a sister taxon of *S*. *plumbea*.

The main objectives of this study are (1) to describe and quantify the source parameters of clicks produced by *S*. *plumbea* and *T*. *aduncus* in Menai Bay, Zanzibar, and (2) to investigate whether there are sufficient differences in the acoustic click parameters of *S*. *plumbea* and *T*. *aduncus* to classify recorded clicks to species.

## Materials and methods

### Data collection

Data were collected on 19 and 20 January, and between 28 June and 19 August 2015 in the Menai Bay Conservation Area, off the southwest coast of Unguja Island, Zanzibar (Fig 1). The study area was surveyed for dolphins using an outboard powered 8 m boat during daytime in Beaufort Sea state <4. The water depth at the recording locations was between 10 m and 15 m depending on the tide. The seabed sediment was sand with scattered small coralline rocks.

The time, date, location, species, group size and surface behavior were recorded for all encountered dolphin groups. The boat motored slowly ahead of the dolphin group, deployed the recording equipment, turned off the engine and drifted to reduce background noise. All acoustic recordings were made using a single SoundTrap (ST) 202HF (Ocean Instruments, New Zealand) with a flat frequency response from 20 Hz to 150 kHz (±3 dB). Full-scale responses and sampling rates were set as 173 dB re 1 μPa and 576 kHz, respectively. The ST has an anti-aliasing filter at 150 kHz, resulting in a– 6 dB roll-off per additional octave in frequency. The ST was deployed approximately 3 m below the sea surface, attached to a surface buoy and a small weight and tethered to the boat by a 50 m floating line. During recordings, dolphins passed or milled within 5–50 m from the boat. There was no apparent reaction by either species to the presence of the boat and the ST. Although some mixed species groups of *S*. *plumbea* and *T*. *aduncus* were encountered and recorded, for the purpose of this study only recordings from single species were used in the analyses. The temporal, spatial and species information for each recording session is given in Table 1.

**Table 1:** Temporal, spatial and species information of acoustic recordings made during 2015 in Menai Bay, Zanzibar, East Africa during single species group encounters of *S*. *plumbea and T*. *aduncus*. Number of click trains, selected trains and individual clicks are presented for each recording session and species.

Species	Recording session	Date	Start time	Latitude (S), Longitude (E)	Group size	Click trains	Selected trains	selected clicks
*S*. *plumbea*	1	19 Jan	2:12 PM	6°27'0", 39°27′36"	5	61	10	10
	2	28 Jun	6:46 AM	6°28'48", 39°29′24"	5	9	9	9
	3	1 Jul	8:21 AM	6°26'24", 39°27′36"	4	3	3	3
	4	20 Jul	12:06 AM	6°27'36", 39°28′12"	8	13	13	13
*T*. *aduncus*	1	20 Jan	7:09 AM	6°27'36", 39°28′12"	25	40	40	40
	2	28 Jun	9:47 AM	6°27'0", 39°27′36"	14	1	1	1
	3	4 Jul	9:26 AM	6°28'48", 39°29′24"	27	4	4	4
	4	17 Jul	7:03 AM	6°28'48", 39°30′36"	5	24	10	10
	5	1 Aug	7:28 AM	6°28'12", 39°32′60"	7	2	2	2
	6	9 Aug	9:07 AM	6°28'12", 39°28′12"	17	2	2	2
	7	11 Aug	8:59 AM	n. a.	36	30	30	30
	8	19 Aug	11:54 AM	6°28'48", 39°28′48"	14	3	3	3

### Data analyses

#### Click train selection

Recordings were first visually and aurally inspected using waveforms and spectrograms [Hanning window, fast Fourier transform (FFT) size: 1024 points, 50% frequency overlap] produced in Adobe Audition (version 3.0, Adobe Systems Incorporated, CA). All sound files were digitally filtered with a 4-order Butterworth band pass-filter (10–200 kHz) in Audition to minimize the influence of whistles and ambient noise. Only sound files with “loud and clear” click trains were extracted, labeled by hand, and used in subsequent analyses. Click trains were considered to be “loud and clear”, if they were at least 10 dB re 1 μPa louder than background noise [40] and had no overlap with other strong pulsed sound. The click trains were inspected and potentially confounding sounds (e.g. snapping shrimp and bubble/sediment entrainment noise) were removed by hand. Furthermore, the chosen click trains were required to contain at least eight clicks per train with average inter-click interval (ICI) of > 10 ms and < 0.1 s using a playback rate of 0.01 [41, 42], thus excluding echolocation buzzes (click trains with high repetition rates used during prey capture) and burst pulses, to avoid introducing additional variance in the dataset. The total number of click trains used per recording session was limited to twice the estimated group size to reduce over-presentation of a single recording session [13]. Click trains were randomly selected until all available trains were selected or the limit was reached (Table 1).

#### Click detection

Clicks were automatically detected from each chosen click train using an energy detector to identify impulse signals [43]. Click trains were first divided into several 5 ms segments. Clicks were detected in the spectra domain (frequency vs spectral power, Hanning window, FFT size: 576 points, 50% overlap) and calculated from each segment. When 13% or more of the frequency bins between 15 kHz and 95 kHz had signal-to-noise ratios over 15 dB [13], the segments were considered to contain a click candidate.

An automated algorithm was used to remove false positive detections, including vessel noise and clipped clicks. Specifically, click candidates with a peak frequency less than 20 kHz [44] and with a maximum amplitude more than 80% of the maximum system capability [14] were considered as false positive detections. Given that only a single hydrophone was used during the field recordings, it was impossible to determine whether a click was recorded on the acoustic axis [45]. To mitigate against the impact of off-axis click use, only the highest amplitude click from each click train was extracted, following the methods for on-axis click analysis [46]. The aforementioned click detections and false positive removal were performed using customized routines in MATLAB (version R2016a, Mathworks, Natick, MA).

#### Acoustic parameters

A 32-point rectangular window around the peak of the signal envelop was utilized for all selected clicks in order to minimize the risk of reflected clicks and background noise being included in the analysis. To assess potential differences in clicks between *S*. *plumbea* and *T*. *aduncus*, eight acoustic parameters, i.e. -10 dB duration, peak frequency, centroid frequency, lower -3 dB frequency, lower –10 dB frequency, -3 dB bandwidth, -10 dB bandwidth and RMS bandwidth, were calculated using custom written scripts in MATLAB R2016a (Mathworks, Natick, MA). The chosen parameters (Table 2) have been used by several other studies to characterize dolphin clicks [7, 14, 46]. The -10 dB duration was determined from the interpolated (10 times linear interpolation) waveform for an individual click. The remaining seven frequency and bandwidth parameters were computed from the power spectra. The power spectrum of each detected click was calculated based on Welch method [47] using 32-point fast Fourier transform with a Hanning window, and interpolated with a factor of 10 using low-pass interpolation, resulting in a spectral resolution of 1.8 kHz. These settings allow direct comparison to previous published acoustic parameter measurements for the two species [23]. Inter-click interval (ICI) and Q (quality factor) parameters were not deemed appropriate for use in the analyses and so were not considered. ICIs are adjusted by the echolocating animal and shortened when approaching a target to facilitate close distance tracking and capture [14]. The Q parameter has been used to describe the relative bandwidth of click signals in previous research [23]. However, the Q parameter does not provide useful information for classification, as it is defined as the ratio of centroid frequency to RMS bandwidth.

**Table 2:** Description of eight acoustic parameters for echolocation clicks of *S*. *plumbea* and *T*. *aduncus* recorded in Menai Bay, Zanzibar, East Africa. Abbreviation used are shown in the parenthesis.

Acoustic parameters	Description
-10 dB duration (D_-10dB_)	Click duration in 10 dB below the peak of the envelope of the waveform [46].
Peak frequency (F_P_)	Frequency value of maximum energy in the spectrum [7].
Centroid frequency (F_C_)	Average power distributed across the frequency bins in the spectrum [48].
Lower -3dB frequency (F_L3_)	Lower cut-off frequency of -3dB bandwidth [14].
Lower -10dB frequency (F_L10_)	Lower cut-off frequency of -10dB bandwidth [14].
-3dB bandwidth (BW_-3dB_)	Frequency width between the 1/√2 of amplitude points of the spectrum on the linear scale [46].
-10dB bandwidth (BW_-10dB_)	Frequency width between the 1/10 of amplitude points of the spectrum on the linear scale [46].
Root-mean-squared bandwidth (BW_RMS_)	Spectral standard deviation around the centroid frequency of the spectrum [48].

### Statistical analyses

The click train selection criteria used identified 35 *S*. *plumbea* (S1 Audio) and 92 *T*. *aduncus* (S2 Audio) click trains as “loud and clear”, and thus suitable for analysis. All data of the highest amplitude clicks selected from each click train used for statistical analysis are available in supporting information (S1 Table). None of the analyzed parameters conformed to a normal distribution (Kolmogorov-Smirnov tests, α = 0.05), had equal variance (Levene’s tests, α = 0.05) or could be successfully log-transformed. Median with 5th and 95th percentile values were used as the descriptive statistics for each parameter. Non-parametric Mann-Whitney U-tests were used to statistically compare the acoustic parameters between *S*. *plumbea* and *T*. *aduncus*. Significance level was set at α = 0.05. All parameters were used in subsequent classification analyses, with parameters showing significant differences between species expected to provide greatest classification power.

Random Forest (RF) was used to separate echolocation clicks between *S*. *plumbea* and *T*. *aduncus* using all acoustic parameters. RF, as a supervised classification method, has demonstrated excellent performance in bio-acoustic studies [49]. The RF is an ensemble classifier, developed by Breiman [50], consisting of many independent classification trees [51], where each tree is generated by a randomly selected subset of the original training data (e.g. 92 *T*. *aduncus* clicks and 35 *S*. *plumbea* clicks used here) with replacement [50, 52]. At each split approximately 37% of the training data, named as “out of bag” (OOB) samples, are not selected when constructing each tree but used to assess the performance of the RF [50, 53]. The remaining samples, named as “in-bag” samples, are used to construct each tree using a random subset of all features (e.g. the eight acoustic parameters here) to split the node [50, 53]. Once the forest is built, individual trees are combined through a majority voting process to assign new candidates to a class [50].

The OOB error rate (OOB_error rate_) was calculated as the median of the error rates from the constructed trees in the RF using OBB samples [50]. The percentage, i.e. 1- OOB_error rate,_ and its 5th and 95th quantiles are reported as a measure of the correct classification rate [54]. There are two significant parameters in the RF [53]: (1) the number of trees to construct (n_tree_), and (2) the number of randomly chosen variables (e.g. acoustic parameters in this case) used to split each node (m_try_), which can be optimized via the OOB error estimation. As a result, the n_tree_ and m_try_ were set to 3000 and √M based on the OOB error, where M represents the total number of input variables.

The above parameter comparison and RF model construction were all implemented in R (version 3.3.3, R Core Team, 2018) using the *asht* and *randomForest* packages, respectively.

### Ethics statement

The research was undertaken with research permits obtained through the Zanzibar Ministry of Agriculture, Natural Resources, Livestock and Fisheries as well as ethical approval from Newcastle University, UK.

## Results

**Fig 2:**
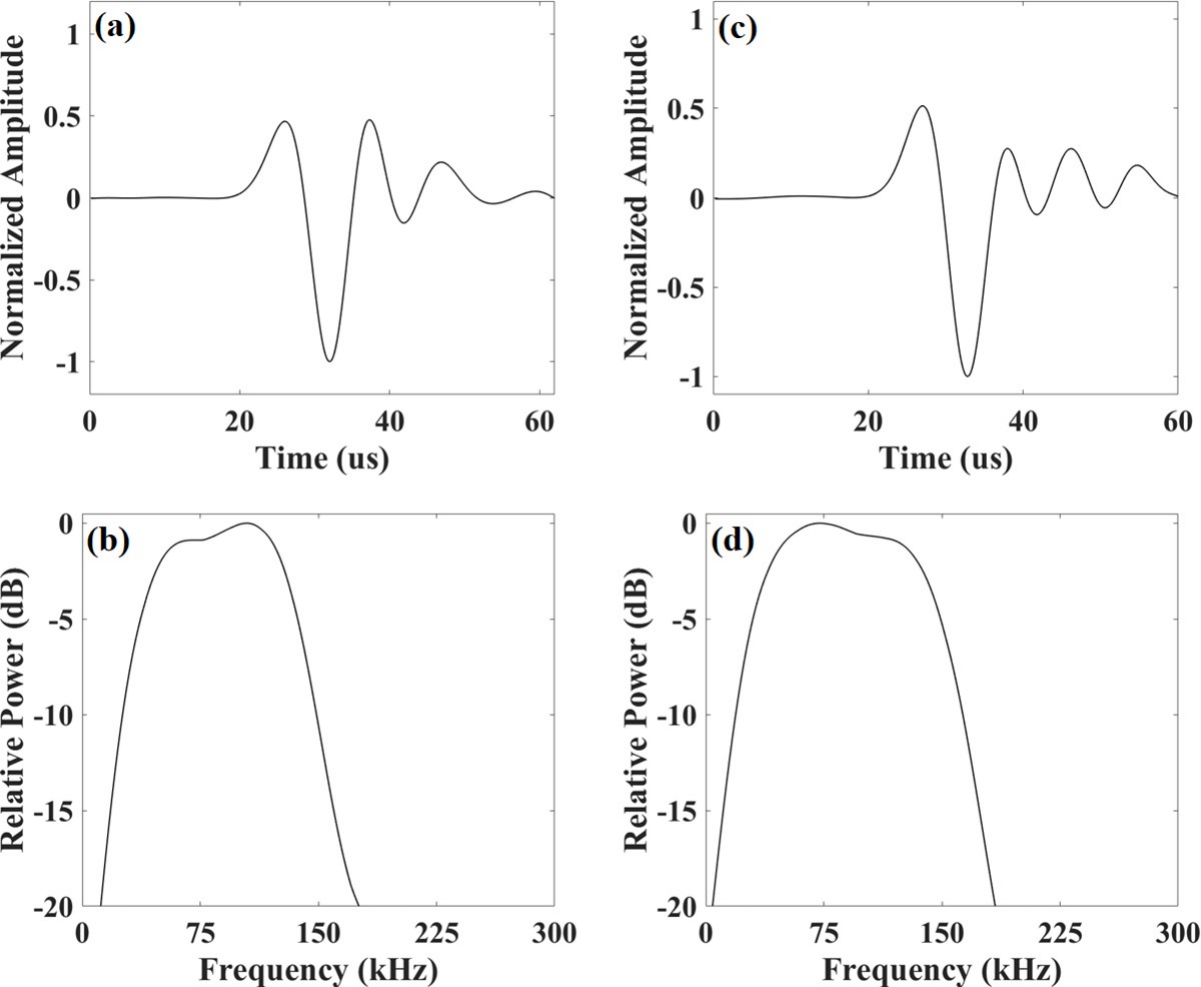
Echolocation click examples for *S*. *plumbea* and *T*. *aduncus*. Waveform and power spectrum [Sampling rate: 576 kHz, 32-point rectangular window around the peak of the envelope, and interpolated with a factor of 10 for a spectral resolution of 1.8 kHz] examples of echolocation clicks of *S*. *plumbea* [(a) and (b)] and *T*. *aduncus* [(c) and (d)] from Menai Bay, Zanzibar, East Africa.

The values of the eight acoustic parameters of *S*. *plumbea* and *T*. *aduncus* echolocation clicks are summarized in Table 3 and their respective typical waveforms and the power spectra are presented in Fig 2. Although the clicks of *S*. *plumbea* and *T*. *aduncus* were similar, the four frequency parameters (peak frequency, centroid frequency, lower -3 dB frequency and lower -10 dB frequency) had significantly higher median values for *S*. *plumbea* compared to *T*. *aduncus* (Mann-Whitney U Tests, α = 0.05, Table 3). Neither -10 dB duration nor the three bandwidth parameters were significantly different between *S*. *plumbea* and *T*. *aduncus*.

**Table 3:** The median with the 5th and 95th percentile values of eight acoustic parameters for echolocation clicks of *S*. *plumbea* and *T*. *aduncus* in Menai Bay, Zanzibar, East Africa. Species comparisons of click parameters were conducted using Mann-Whitney U tests. Note: * = p<0.05.

Acoustic parameters	*S*. *plumbea* (n = 35)		*T*. *aduncus* (n = 92)		p-value
	median	(5–95%)	median	(5–95%)	
-10 dB duration (μs)	13	(11–22)	14	(11–23)	0.514
Peak frequency (kHz)	97	(52–119)	73	(47–115)	0.003*
Centroid frequency (kHz)	87	(64–107)	81	(57–98)	0.002*
Lower -3 dB frequency (kHz)	43	(29–95)	36	(27–59)	0.003*
Lower -10 dB frequency (kHz)	22	(16–35)	18	(13–29)	0.017*
-3 dB bandwidth (kHz)	81	(49–107)	85	(34–95)	0.223
-10 dB bandwidth (kHz)	126	(103–148)	130	(94–146)	0.445
RMS bandwidth (kHz)	23	(18–31)	25	(16–35)	0.365

Overall, the RF model resulted in a 73.2% (5th and 95th quantiles 72.4%-74.0%) correct classification rate for *S*. *plumbea* and *T*. *aduncus* echolocation clicks submitted to the model, compared to 50% expected by random chance alone. Similarly, the RF model resulted in a correct classification rate of 28.6% (5th and 95th quantiles 25.7%-31.4%) for *S*. *plumbea*, compared to an expected rate of 27.6%, and 90.2% (5th and 95th quantiles 90.2%-90.2%) for *T*. *aduncus*, compared to an expected rate of 72.4%. The RF model demonstrated the following order of importance for the acoustic parameters using mean decreasing accuracy measures for species classification: lower -10 dB frequency, -3 dB bandwidth, -10 dB bandwidth, lower -3 dB frequency, centroid frequency, RMS bandwidth, peak frequency and -10 dB duration.

## Discussion

To our knowledge, this study represents the first report of echolocation click parameters for *T*. *aduncus* in the Western Indian Ocean and for *S*. *plumbea* globally. The study found differences in peak, centroid, lower -3 dB and lower -10 dB frequencies of clicks between species. This study further classified the echolocation clicks using a RF model and achieved an overall 73.2% correct species classification rate i.e. a better performance than what was expected by chance alone (50%).

Click source parameters measured in this study differed substantively in some instances from those previously reported for *T*. *aduncus* and *Sousa* species in other geographic regions (Table 4). Both species had lower peak and centroid frequencies than previously reported in the published literature [23, 41, 55]. These differences may result from morphological species differences [14] and/or optimization of clicks for the specific environmental context of different habitats primarily occupied by the species [17]. Indeed, *S*. *plumbea* has a relatively longer and narrower skull compared to *S*. *chinensis* [56]. Additionally, differences among study methodologies may contribute to click parameter differences. The present study used a single hydrophone and selected only the highest amplitude click in each click train for inclusion in the analyses, in order to minimise the likelihood of off-axis click selection. Other studies were able to use hydrophone arrays thus having a higher likelihood of selecting only on-axis clicks [23, 55].

**Table 4:** Comparisons of the mean ± standard deviation of source parameters of echolocation clicks of wild *T*. *aduncus* from different areas and different species of *Sousa*.

Species	-10 dB duration (μs)	Peak frequency (kHz)	Centroid frequency (kHz)	-3 dB bandwidth (kHz)	-10 dB bandwidth (kHz)	RMS bandwidth (kHz)	Reference
*T*. *aduncus* (n = 54)	14±2	124±13	112±9	62±17	140±17	34±3	[23]
*T*. *aduncus* (n = 89)	18±6	n.a	91±13	n. a.	n. a.	35±3	[41]
*T*. *aduncus* (n = 92)	16±4	77±24	80±13	81±19	127±15	24±4	This study
*S*. *chinensis* (n = 77)	19±4	109±4	95±6	50±13	102±11	29±3	[55]
*S*. *sahulensis* (n = 42)	15±2	114±12	106±11	59±18	116±20	29±4	[23]
*S*. *plumbea* (n = 35)	16±3	91±22	87±12	79±15	125±11	24±3	This study

Statistically, all click frequency parameters (peak, centroid, lower -3 dB and lower -10 dB frequencies) measured for *S*. *plumbea* were higher than those of *T*. *aduncus*. Differences in preferred habitat and cranial morphology likely explain the interspecific click differences observed in these parameters. *T*. *aduncus* and *S*. *plumbea* in Menai Bay have overlapping distributions. However, *S*. *plumbea* is only found in shallow waters, close to shore whereas *T*. *aduncus* occurs across the bay including offshore areas [30]. The significantly lower echolocation click frequencies recorded for *T*. *aduncus* compared to *S*. *plumbea* would facilitate longer range echolocation which would be beneficial in the deeper and more open water habitats occupied by *T*. *aduncus* [57]. Furthermore, differences in skull morphology between species, with *S*. *plumbea* featuring “a small left posterior branch of the melon”, which may be an adaptation that provide improved directionality when using high frequency sounds [57].

The parameters used for species classification in the present study were similar to those applied in other similar research [11, 14, 23]. Specifically, frequency parameters appear to be powerful for classification of delphinids from clicks. Bandwidth parameters appear, generally, weaker for classification, which is consistent with findings of no or limited differences in these parameters among species [14, 23]. Despite this, bandwidth parameters were found to be relatively important contributors to species classification in this study, suggesting that researchers should continue to consider these parameters in future. Interestingly, whilst none of the bandwidth parameters showed significant differences between the two species, both the -3 and -10 dB bandwidth parameters were important in the RF classifier. This reflects the multivariate nature of click signals and indicates likely interactions among parameters. It re-enforces the importance of retaining parameters for classification despite not showing significant differences across all clicks. Conversely, click duration contributed little to click classification. This is congruent with the limited variability in this parameter for sympatric *Tursiops* and *Sousa* species observed in this study and other studies [23]. However, it is notable that click duration has been identified as a valuable parameter for classification of other delphinid species [12, 58] and thus cannot be assumed irrelevant in future works. Understanding drivers behind the interspecific variability in parameter utility for classification may be an important step in shaping future classifier development and ultimately its field application.

Regarding classification of *T*. *aducus* and *S*. *plumbea* in the current study, both species were classified successfully above the expected rate. However, the improvement above the expected rate for *S*. *plumbea* was minimal. The sample size for *S*. *plumbea* was relatively small (n = 35) in comparison to *T*. *aduncus* (n = 92) and several of the parameters showed a high level of variability. The relatively small sample size for *S*. *plumbea* makes it vulnerable to influence from unusual click trains or accidental inclusion of off-axis trains. Therefore, greater sampling effort is required to confirm and improve on the findings in the current study. The relatively high classification rate for *T*. *aduncus* demonstrates the potential to use PAM of clicks to monitor occurrence of this species. Thus, continued effort to further improve the classifiers and to develop algorithms to be incorporated into PAM monitoring systems for *T*. *aduncus* is strongly encouraged.

In this study, eight acoustic parameters were considered in the analysis. Future efforts to classify species using clicks may be assisted by expanding the range of parameters considered [59, 60]. For example, measurements of intensity at different frequencies in the spectrum have been shown to facilitate identification of “peak and notch” patterns which have been used to classify both *G*. *griseus* and *L*. *obliquidens* [13]. Furthermore, comparing intensity at different frequency bands in the spectrum has improved classification results for *M*. *densirostris*, *G*. *macrorhynchus* and *G*. *griseus* [61, 62]. Previous research have also demonstrated that coefficients in cepstral analysis (inverse Fourier transform of the logarithm of the estimated spectrum of a signal) had good classification performance for some species (e.g. *G*. *griesus*, *M*. *densirostris* and *G*. *macrorhynchus*), with little influence of sound propagation and variation resulting from different recording platforms [15, 63]. Additionally, energy amplitude, off-axis click distortion and transmission beam-widths may contain some species-specific information. However, these characteristics are dependent on knowing the exact location and orientation of echolocating individuals which requires a multiple hydrophone array which was not available in the present study.

In conclusion, this study presents new information on echolocation click parameters recorded from wild *S*. *plumbea* and *T*. *aduncus* resident in Menai Bay, Zanzibar. We further explored whether variation in acoustic parameters of echolocation clicks may be used to identify and classify sympatric living *S*. *plumbea* and *T*. *aduncus*. An overall 73.2% species click classification rate was achieved, indicating the potential to separate these two species using PAM. However, the identified classifiers were only at a sufficiently high rate (90.2%) for *T*. *aduncus* to allow species specific monitoring using PAM based on echolocation clicks. The classification method developed would benefit from further refinement and may be improved by increasing the suite of acoustic parameters considered. It is anticipated that the methods eventually can be incorporated into PAM systems as species-specific classification algorithms, facilitating the use of PAM methodologies to monitor occurrence of specific delphinid species. Such information would provide researchers and managers with the foundational data needed to devise evidence-based species-specific conservation strategies, particularly in areas where species are threatened by anthropogenic activities.

## Supporting information

S1 Audio Echolocation click trains of Indian Ocean humpback dolphin (*Sousa plumbea*). (ZIP)

S2 Audio Echolocation click trains of Indo-Pacific bottlenose dolphin (*Tursiops aduncus*). (ZIP)

S1 Table Information for each click analyzed. For each click, information is provide indicating species, file name, -10 dB duration (μs), peak, centroid, lower -3 and lower -10 dB frequencies (kHz), and -3 dB, -10 dB and root-mean-squared bandwidth (kHz).
(XLSX)
